# Long-duration head down bed rest as an analog of microgravity: Effects on the static perception of upright

**DOI:** 10.3233/VES-210016

**Published:** 2022-07-22

**Authors:** Laurence R. Harris, Michael Jenkin, Rainer Herpers

**Affiliations:** a Centre for Vision Research, York University, Toronto, Canada; b Department of Psychology, York University, Toronto, Canada; c Department of Electrical Engineering and Computer Science, York University, Toronto, Canada; d Institute of Visual Computing, Bonn-Rhein-Sieg University of Applied Sciences, St. Augustin, Germany

**Keywords:** Human orientation perception, space flight analog, subjective visual vertical, perceptual upright, head down bed rest, HDBR

## Abstract

**BACKGROUND:**

Humans demonstrate many physiological changes in microgravity for which long-duration head down bed rest (HDBR) is a reliable analog. However, information on how HDBR affects sensory processing is lacking.

**OBJECTIVE:**

We previously showed [25] that microgravity alters the weighting applied to visual cues in determining the perceptual upright (PU), an effect that lasts long after return. Does long-duration HDBR have comparable effects?

**METHODS:**

We assessed static spatial orientation using the luminous line test (subjective visual vertical, SVV) and the oriented character recognition test (PU) before, during and after 21 days of 6° HDBR in 10 participants. Methods were essentially identical as previously used in orbit [25].

**RESULTS:**

Overall, HDBR had no effect on the reliance on visual relative to body cues in determining the PU. However, when considering the three critical time points (pre-bed rest, end of bed rest, and 14 days post-bed rest) there was a significant decrease in reliance on visual relative to body cues, as found in microgravity. The ratio had an average time constant of 7.28 days and returned to pre-bed-rest levels within 14 days. The SVV was unaffected.

**CONCLUSIONS:**

We conclude that bed rest can be a useful analog for the study of the perception of static self-orientation during long-term exposure to microgravity. More detailed work on the precise time course of our effects is needed in both bed rest and microgravity conditions.

## 1 Introduction

Long-term exposure to microgravity is known to adversely affect a wide range of physiological systems including bone density [49], cardiovascular performance [10], and eye structure ([31] see [43, 45, 50] for reviews). These physiological effects are also coupled with changes in sensorimotor functions [8] including gait post flight [7, 40], gaze [38] and changes in perception associated with vestibular function and its interaction with other sensory systems (see [24, 41] for reviews), including the perception of static [25] and dynamic [16] self-orientation (see [11] for a review). The study of the effects of microgravity on humans and their performance is limited by the risk, cost, and operational difficulties associated with deploying participants and test equipment in a microgravity environment and by the limited time available for such tests for astronauts working on orbit. One potential alternative for conducting studies on the effect of removing gravity from the long axis of the body is through head down long-duration bed rest (HDBR). HDBR has been used extensively as an analog for microgravity although there are well-known differences between the physiological changes associated with spaceflight and HDBR [45]. However, whether HDBR leads to perceptual effects such as are found following long-term exposure to microgravity is unknown.

In addition to the physiological changes associated with long-duration spaceflight, many aspects of perception are also altered in microgravity. Visual reorientation illusions and motion sickness are regularly reported in weightlessness [42, 46, 47]. The perception of self-orientation is also altered [13, 24] and the perceived tilt of the body is overestimated immediately on return to earth [14–17]. Not only are many of these perceptual effects debilitating, but they also represent a significant safety hazard when navigating in an emergency, and operational issues when working with oriented switches and devices. Only a few studies have attempted to look at whether HDBR induces comparable perceptual effects and they have generally had disappointing results [27, 39]. Here we investigate the effects of HDBR on the perception of self-orientation using a method that has proven successful in long duration spaceflight.

Self-orientation refers to our perceived orientation relative to an external frame. On Earth this reference frame is usually gravity where the perception is derived from visual and physical cues concerning the direction of gravity relative to the body from which an internal representation of this orientation is created and constantly updated [32] even in the absence of vision. The direction of gravity relative to the body can be inferred from the vestibular apparatus combined with knowledge of the orientation of the head on the body, signalled by neck proprioceptors [12, 23, 51]. The direction of gravity relative to the body is also indicated by the somatosensory system that registers pressure on the skin where it touches a support surface [2] and from specialized organs in the mesentery of the kidneys [34]. Mittelstaedt [33] postulated that the perceived vertical is determined by a combination of an idiotropic vector (an internal representation of the body axis), the gravitational vertical (obtained from multiple sources), and visual cues to upright. The classic test for establishing a participant’s perceived direction of up is the luminous line test (LL) [19, 26] which measures the subjective visual vertical (SVV). To obtain an estimate of the SVV, participants adjust the orientation of a line until it is perceived as aligned with gravity. Using the LL test to measure how the SVV varies with body tilt and alterations in the sensory environment has a long history since its accidental discovery by Aubert [4]. The SVV is affected by clinical damage to the vestibular apparatus [20], stroke [55] and cortical damage [18]. When Moore et al. [39] compared the SVV before and after HDBR they found no effect on the errors regularly found in the SVV [4] when measured with the body rolled 90°. This matched the lack of changes found in the SVV after spaceflight [28]. However, the LL test suffers from important limitations: First, the LL test cannot be used when there is no gravity direction with which to align the line. Second, while the SVV is influenced by factors other than gravity [54], vision plays a relatively small role [21, 35, 53]. The SVV is therefore an insensitive measure of the relative contributions of vision and gravity to self-orientation. Third, the SVV introduces cognitive factors, including cueing the participant about the specific purpose of the task, because it requires participants to consciously examine their perceived “up” direction.

Given these limitations of the LL test, we developed an alternative measure of perceived self-orientation known as the Oriented Character Recognition Test (OChaRT) [21] for use in microgravity [25]. This test identifies the orientation at which an ambiguous character (e.g., the letter “p”) whose interpretation depends on its orientation (e.g., either a “p” or a “d”) appears least ambiguous. Since all the participant has to do is identify the character, there is no need to make a conscious comparison with gravity. We refer to the orientation that this test yields as the perceptual upright (PU). By systematically varying the orientation of the visual or body cues to upright relative to gravity by viewing the character against a tilted background and by positioning the participant on their side, the relative contributions of each of these cues relative to constant gravity can be ascertained. Furthermore, visual cues to orientation can be removed by displaying the character against a featureless background, and the influence of gravity can be removed from the plane of testing by lying supine. By using such manipulations, it has been determined that the PU is more evenly influenced by the contributing cues than is the SVV. A typical distribution of the relative contributions of the components is 54%body, 25%vision and 21%gravity for the PU, compared to 15%body, 8%vision and 77%gravity for the SVV [1, 21].

Using OChaRT, Harris et al. [25] demonstrated systematic changes in the perception of the PU during and following long-duration spaceflight. They reported that the ratio of the weightings of visual cues relative to body cues that determine the PU increased when tested early during spaceflight. This effect disappeared later in flight but re-appeared a few weeks after return to a 1G environment. Harris et al. [25] also demonstrated that the variability of the SVV increased after long-duration spaceflight. If HDBR were a suitable microgravity analog for the perception of self-orientation, then it should elicit similar effects. We therefore performed OChaRT and LL before, during, and after HDBR to see if the effects we observed associated with long-duration microgravity exposure were also found during HDBR. In order to assess whether HDBR is an effective analog for the effect of microgravity on the perception orientation, we compared the effect of visual and body roll on the PU and SVV before and after 21 days of bedrest. Taking advantage of the ability to use OChaRT effectively during bedrest, we also assessed the time course of any effects on the PU at days 7, 14 and 21 during the bedrest experience. Our HDBR data were then compared with corresponding data collected during long duration space flight [25].

The integration of multisensory cues to produce a single percept (in this case self-orientation) can be modelled by a weighted average in which the weightings are assigned in proportion to the reliability of the cues involved [22]. Any change from this statistically optimal process in generating the perception of self-orientation would imply that additional context-dependent processes (in this case, being in microgravity) are able to affect the process of multisensory integration.

## 2 Methods

The effect of an oriented visual scene and changes in body postures on the subjective visual vertical and perceptual upright were assessed before, during and after 21 days of 6° head down bed rest. The subjective visual vertical was measured by means of a luminous line probe and the perceptual upright was measured using an ambiguous character (“p/d”). Body orientation was varied between supine, lying on the side and, before and after the bed rest experience, upright.

### 2.1 Participants

Ten male, native German-speaking participants (mean age 31 years, SD±6 years, range 23–42 years) participated in the bed rest study. One failed to complete the bed rest paradigm. All had normal or corrected-to-normal vision. None reported any history of vestibular disease or damage. Participants were pre-screened for a wide range of physiological and psychological factors to ensure that they could deal with the stresses associated with long-duration bed rest. Medical staff at the bed rest facility monitored participants’ health continuously. All participants signed a written consent form. The York University Research Ethics Board Committee as well as the ethics commission of the Ärztekammer Nordrhein (Düsseldorf, Germany) approved the experiments. The experiments followed the principles laid down in the Declaration of Helsinki.

There were two HDBR sessions, each of 21 days duration, separated by three months. During HDBR, participants were subjected to a range of biological and perceptual experiments as well as ours, although all experiments were carefully controlled to ensure that the participant moved as little as possible from a 6° head down body orientation. As part of a wider protocol, our participants were subjected to an intervention regime that was primarily intended to investigate the effect of different diets and supplements on bone and tissue damage associated with long-duration bed rest. Further details of the general methods associated with the HDBR procedure are described by Buehlmeier et al. [9]. Participants received normal or intervention diets depending on the bed-rest session, with half of the participants receiving the intervention in the first session and the other participants receiving the intervention in the second session. The intervention involved replacing fat and carbohydrates in the participants’ diet with a whey protein supplement to alter their acid-base balance as changes in this balance are suspected to reduce bone loss associated with microgravity flights and long-duration bed rest [56]. It was not anticipated that this intervention would influence the participants’ perception of self-orientation.

Due to the restricted size of the individual rooms of the bed rest facility and the arrangement of the bed in each room, it was not possible to set up our monitoring equipment on both sides of each bed. Therefore, one half of the participants were tested in bed supine (6° head down) and left side down (6° head down left side), while the other half were tested in bed supine (6° head down) and right side down (6° head down right side).

### 2.2 Equipment

**Fig. 1:**
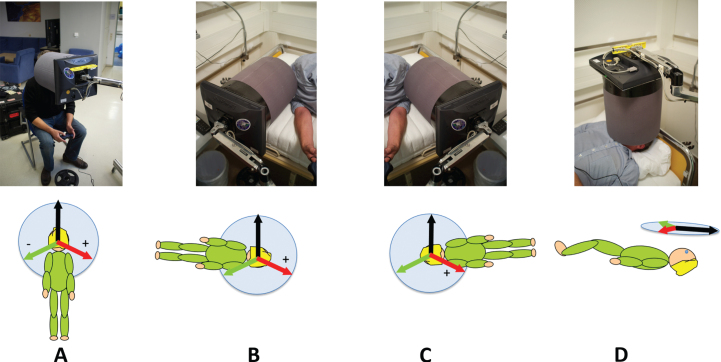
The body orientations and equipment used in this study. The viewing tube and monitor isolated the participant’s view of stimuli from the outside world (A) upright, (B) right side down (RSD), (C) left side down (LSD), and (D) supine. Beneath each photograph is a diagram indicating the orientation of the three visual backgrounds used.

Stimuli were displayed on an LCD panel (ViewSonic VA705B 1280x1024 resolution) connected to an Apple Laptop (Apple MacBook Pro) which was viewed through a cylindrical tube (diameter 19 cm, length 21 cm) made of black foam (Fig. 1). The field of view was 48°. Viewing distance was 21 cm, controlled by the length of the viewing tube. Participants responded using a USB gamepad (Gravis Gamepad Pro). The tube and screen were mounted together on a device that could be arranged so that participants could view the screen sitting up (Fig. 1A), while lying on their left or right sides (Fig. 1B and C) or while lying supine (Fig. 1D).

### 2.3 Stimuli

**Fig. 2:**
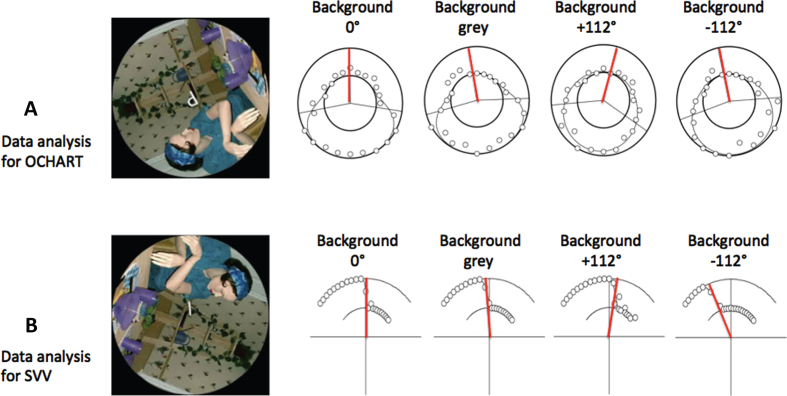
Sample responses for the SVV and OChaRT probes. Each row shows responses for one participant for the four backgrounds for a single body orientation. The figure in the visual display is a photograph of a manikin added to provide realistic orientation cues. (A) OChaRT: A product of two psychometric functions is plotted through the data in polar coordinates where the outer circle represents “probe interpreted as a ‘d’ 100%of the time” and the inner circle represents “probe interpreted as a ‘p’ 100%of the time”. The PU is defined as the midpoint between the PSEs (50%points) of the two psychometric functions, indicated by red radial lines. (B) SVV: A psychometric function is plotted through the data in polar coordinates where the outer circle represents “probe tilted to the left of gravity 100%of the time” and the inner circle represents “probe tilted to the right of gravity 100%of the time”. The SVV is the 50%point of this curve, indicated by the red radial lines. Sample OChaRT and SVV stimuli are also shown.

All probes were presented in front of a visual background that was either grey (no visual cues to upright) or a rotated version of a highly polarized scene with many visual cues to the direction of gravity that was displayed either upright or rotated±112° relative to the gravitational vertical, except for the supine viewing condition when “upright” was defined as being aligned with the body axis (see left hand side of Fig. 2).

### 2.4 Measuring the subjective visual vertical

The subjective visual vertical (SVV) was measured by means of a luminous line (LL) test superimposed on the backgrounds described above (Fig. 2B). The line measured 2.7° x 0.4° and radiated out from a dot (diameter 0.4°) in the centre of the screen. The participant was required to judge whether the line was tilted to the left or right of gravity (the direction in which a ball would fall) and respond with button presses on the gamepad accordingly. The probe was presented for 500msec and then the display was replaced with a grey background and a circular fixation marker. This display remained until the participant responded. For each body orientation, each probe was presented against one of the four backgrounds (see Fig. 1). The luminous line probe was presented at 23 different orientations in 5° increments from –55° to+55° inclusive relative to gravity. Each line probe was presented 7 times for a total of 23 x 7 x 4 = 644 trials per body orientation. Each trial took about 2s so that each condition took about 20mins.

### 2.5 Measuring the perceptual upright

The perceptual upright (PU) was measured by means of the oriented character recognition test (OChaRT) [21]. Here, we used the ambiguous character “p” which appears as a “d” when rotated by 180°. The character measured 1.9° x 3.0° when viewed at the viewing distance of 21 cm. The probe was presented at one of several orientations (see below) and the participant’s task was to indicate if it appeared to be a “p” or a “d”. The orientations at which it appeared most ambiguous were assessed from which the perceptual upright, defined as the orientation midway between these most-ambiguous orientations, was calculated (see Fig. 2). The probe was presented for 500 msec and then the display was replaced with a grey background and a circular fixation marker. This display was presented until the participant responded. For each body orientation, the probe was presented against the same four backgrounds as used in the SVV experiment (Fig. 2A). The ambiguous letter probe was presented every 15° (24 different orientations). Each probe was presented 7 times for a total of 24 × 7 × 4 = 672 trials per body orientation. Sample participant responses to both the LL test and OChaRT probes are illustrated in Fig. 2.

### 2.6 Procedure

**Fig. 3:**
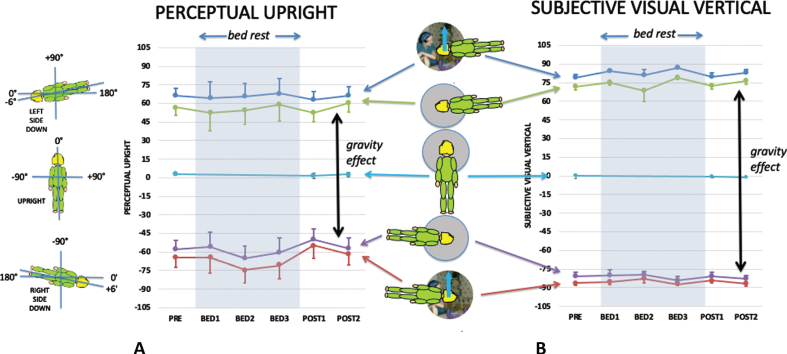
The effect of body posture and visual cues aligned with gravity on the PU (a) and SVV (b) before, during and after bed rest. The bed rest period is indicated by grey shading. The timings of the data collection sessions are given in the methods section under procedure. The mean data are plotted in floor co-ordinates as indicate on the left of the figure so that when left side down (blue and green lines) the PU and SVV are displaced clockwise (positive values) and when right side down (red and purple lines) they are displaced counterclockwise (negative values). Note that *n* = 9 for pre- and post-conditions, but *n* = 4 or 5 for in-bed conditions. The difference between responses with the grey, featureless background during left and right side down is defined as the gravity effect (indicated by the two-headed black arrows) since the only difference in these conditions is the direction of gravity relative to the body. Standard error bars are shown.

**Fig. 4:**
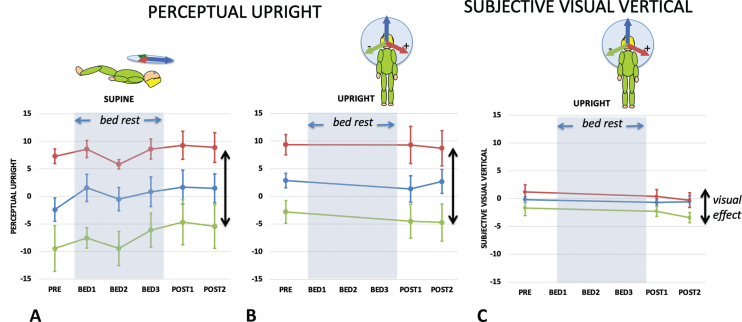
The effect of vision on the PU while supine (a) and upright (b), and on the SVV while upright (c) before during and after bed rest. The bed rest period is indicated by grey shading. The difference between the PU or SVV when vision is orientated to the left and right is defined as the visual effect (indicated by two-headed arrows) since the only difference in these conditions is the direction of the visual cue. Standard errors are shown.

Data were collected over two 21-day bed rest sessions separated by a three-month period with the same participants participating in both sessions. For each session data were collected once during the 14-day period prior to bed rest, twice during the 14-day period post bed rest, and 3 times during bed rest itself (on days 7, 14 and 21). During each of the pre- and post- bed rest, data collection sessions, data were collected with the participant upright (UPR), supine (SUP), left side down (LSD), and right side down (RSD) in a randomized order. During bed rest, data were collected supine, and with the participant either left side down or right side down (both supine and on-side for the PU and just on-side for the SVV). The conditions tested and their sequence can be seen in the inserts for Figs. 3 and 4. For a given condition (e.g., supine in bed) each probe was run as a block with all four visual background orientations (0°,+112°, –112°, and GREY) randomly interleaved. Thus, there were eight blocks (4 orientations x 2 probes) for the pre- and post- bed rest data collections and three blocks for the in-bed data collections (supine and on-side for the PU probe, and just on-side for the SVV probe). The order of blocks was randomized between subjects and between sequential measurements.

### 2.7 Data analysis

#### 2.7.1 Convention

Positive angles are clockwise from the participant’s point of view. All data are described here in either (i) a floor-based coordinate system (with the up direction defined by gravity being at+90° for left-side down conditions and at –90° for right-side down conditions, as illustrated on the left side of Fig. 3), or (ii) for supine conditions a body-based coordinate system where the body midline was defined as 0°.

#### 2.7.2 The subjective visual vertical

For each background, responses were plotted as the frequency with which they chose: “tilted left”. The data were fit with a hyperbolic tangent, which is similar to a cumulative Gaussian sigmoid:

(1) $$\mathrm{fit}\left(x;x_{0},t\right)=0.5*\left(1+\tanh\left(\left(x-x_{0}\right)/t\right)\right)$$

where x is each orientation of the line that was tested. These fits measured not only the orientation of the SVV (x_0_) but also gave an estimate of the variability of the task (t). The hyperbolic tangent sigmoid function was converted to a corresponding Gaussian function and the standard deviation computed for this function. The variance is defined as the square of the standard deviation.

#### 2.7.3 Perceptual upright

The orientation of the PU was determined by fitting a double psychometric function to the frequency with which they chose “p”. The data were fit with a product of two hyperbolic tangents with a common scale factor (t). The PU was defined as halfway between the two points of maximum ambiguity (x_0_ and x_1_). As with SVV, t provides an estimate of the variability of the participant’s response:

(2) $$\begin{array}{cc} & \mathrm{Fit}\left(x;x_{0},x_{1},t\right)\;=\;0.5\;*\;(1-\tanh\left(\left(x-x_{0}\right)/t\right)\;*\\ & \;\;\;\;\;\;\;\;\;\;\;\;\;\;\;\;\;\;\;\;\;\;\;\;\tanh((x-x_{1})/t)) \end{array}$$

Sample results for the PU and SVV along with their fits are given in the Fig. 2.

#### 2.7.4 Data analysis

SVV and PU measurements were computed for each participant in each combination of visual and physical orientations for each condition. Data were collapsed across diet treatments resulting in complete data sets for nine participants. SVV and PU responses for supine and upright postures were used to assess the effect of the visual background. Individual participant responses were then fit to a linear weighted vector sum model and the ratio of visual to body weights were used to assess the effect of HDBR on the perception of self-orientation. The data were analyzed using SPSS 27. Repeated measures ANOVA’s were used as the primary statistical test. Tests that violated sphericity had their degrees of freedom corrected using Greenhouse-Geisser when appropriate. Post-hoc t-tests were performed using Bonferroni correction.

#### 2.7.5 Missing data and collapsing across intervention

Participants were subject to intervention diets with half receiving intervention in the first session and half in the second. Given the long period of data collection it was unavoidable that some data would not be collected, and that participant health would impact data collection. Table 1 summarizes the missing data. Participant E was unable to complete the study for health reasons. For the other participants with missing data, for the statistical analysis of intervention only, the missing data were replaced with that participant’s average response for the same intervention, body orientation, and visual background, and a repeated measures ANOVA performed to examine any effect of intervention. A repeated measures analysis of OChaRT responses for intervention (2: intervention, no intervention) x session (3: pre, post1, post2) x body orientation (4: upright, left side down, right side down, supine) x background (4 : 0°,+112°, –112°, grey) found no effect of intervention F(1,8) = 2.847, *p* = .130, n.s., ɛ_p_^2^ = 0.262. Similarly, a repeated measures analysis of SVV responses for intervention (2: intervention, no intervention) x session (3: pre, post1, post2) x body orientation (3: upright, left side down, right side down) x background (4 : 0°,+112°, –112°, grey) found no effect of intervention F(1,8) = 0.289, *p* = 0.605, n.s., ɛ_p_^2^ = 0.035. The PU and SVV data were therefore collapsed over treatment. This enabled the missing subject responses used in the above analysis to be replaced by either the mean subject response or the subject response under the one available treatment, except for subject E who was dropped from further processing, leaving a subject pool size of nine.

**Table 1:** Summary of missing data. Participant E became ill and was unable to complete the second study and was dropped from the study. All other missing conditions are listed above. For example, participant C missed data in Session 1 in two phases: in Bed Rest Session 1 in the left side down (LSD) body pose with the LL probe in all four visual backgrounds, and in Post Bed Rest Session 1 he missed the right side down (RSD) body pose with the OChaRT probe tested against all four visual backgrounds. All the missing data except for participant E were associated with the first session and thus upon collapse of the dataset over session, there were no missing data

Participant	Session	Phase	Body	PROBE	Background(s)
C	1	BED1	LSD	LL	+112, –112, 0, GRY
C	1	POST1	RSD	OChaRT	+112, –112, 0, GRY
E	1	BED2	LSD	LL	+112, –112, 0, GRY
E	1	POST1	LSD	OChaRT	+112, –112, 0, GRY
E	2	Entire dataset
G	1	PRE	LSD	OChaRT	+112, –112, 0, GRY
G	1	PRE	UP	OChaRT	+112, –112, 0, GRY
G	1	PRE	RSD	OChaRT	+112, –112, 0, GRY
H	1	BED3	RSD	LL	+112, –112, 0, GRY
H	1	BED2	SUP	OChaRT	GRY

## 3 Results

### 3.1 SVV and PU

Figure 3 shows the influence of body orientation and visual cue orientation on the mean PU (Fig. 3A) and the mean SVV (Fig. 3B) throughout the experiment plotted relative to the gravity-defined horizontal. Upright defined by gravity would be 0° for upright and±90° for side down conditions. Body-defined performance would be±90° for side down performance pre- and post-bed rest, and±96° for in-bed conditions (see insert on left of Fig. 3). The visually defined upright provided an additional cue to the direction of up signaled by gravity (solid blue arrows in figure inserts).

### 3.2 Visual and gravity effects

Figure 4 shows the influence of vision on the PU while supine (Fig. 4A) and upright (Fig. 4B), and on the SVV when upright measured before and after HDBR (Fig. 4C). Repeated measures analysis of the effect of bedrest was performed for both upright and supine data for PU and upright only for SVV.

PU Supine: A repeated measures analysis of OChaRT responses was conducted for session (6: pre, bed1, bed2, bed3, post1, post2) x background (3 : 0°,+112°, –112°). Mauchly’s test of sphericity was violated for session χ^2^(14) = 31.892, *p* = .007 and for background χ^2^(2) = 7.210, *p* = .027. DOF for session and background were corrected using Greenhouse-Geisser. There was no effect of session F(2.619, 20.955) = 1.195, *p* = .332 n.s., ɛ_p_^2^ = 0.130, there was an effect of background F(1.217,9.738) = 25.330, *p* < .001, ɛ_p_^2^ = 0.760, and there was no interaction effect F(10,80) = 0.707, *p* = .715 n.s., ɛ_p_^2^ = 0.081.

PU Upright: A repeated measures analysis of OChaRT responses was conducted for session (3: pre, post1, post2) x background (3 : 0°,+112°, –112°). There was no effect of session F(2, 16) = 0.289, *p* = .753 n.s., ɛ_p_^2^ = 0.035, there was an effect of background F(2,16) = 20.0866, *p* < .001, ɛ_p_^2^ = 0.715, and there was no interaction effect F(4,32) = 0.5483, *p* = .702 n.s., ɛ_p_^2^ = 0.064.

SVV Upright: A repeated measures analysis of SVV responses was conducted for session (3: pre, post1, post2) x background (3 : 0°,+112°, –112°). Mauchly’s test of sphericity was violated for session χ^2^(2) = 7.588, *p* = .023, and DOF for session was corrected using Greenhouse-Geisser. There was no effect of session F(1.204,9.628) = 1.189, *p* = .315 n.s., ɛ_p_^2^ = 0.129, there was an effect of background F(2,16) = 8.786, *p* = .003, ɛ_p_^2^ = 0.523, and there was no interaction effect F(4,32) = 0.713, *p* = .589 n.s., ɛ_p_^2^ = 0.082.

**Fig. 5:**
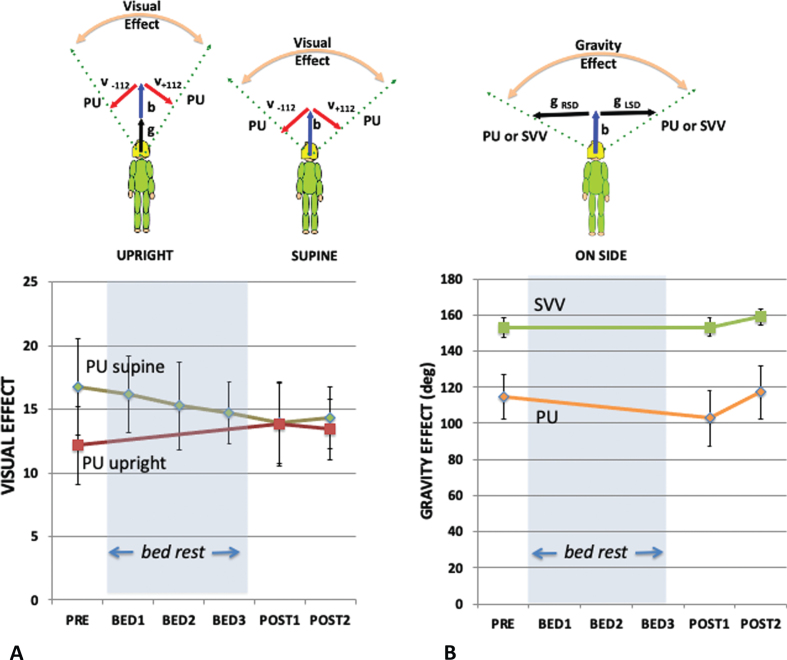
The visual effect (A) and gravity effect (B). (A) The visual effect is the difference in the orientation of the PU or SVV between when the visual background is tilted maximally left (–112) and right (+112) as shown in the cartoon above where the red arrows indicate the visual “up”, the blue arrow the body “up”, and the black arrow the gravity “up”. The dashed arrows indicate the PU or SVV settings. The visual effect (A) is plotted for the PU for each of the five sampling points when supine (green line) or upright (red line). The vector model (above) indicates that the visual effect is expected to be larger when lying supine than when upright. (B) The gravity effect is the difference in the orientation of the PU or SVV between when the participant is tilted so that gravity is directed left (g RSD) or directed right (g LSD). The gravity effect (B) is plotted for the PU (orange line) and SVV (green line) for each of the five sampling points. Standard errors are shown.

The difference in PU caused by altering the visual background (here between +112° and –112°) reveals the effect of vision on the perceived direction of upright while keeping the other cues constant and is referred to as the visual effect (VE). Comparing the VE in the upright and supine conditions is important because the PU in the frontal plane when upright is determined by vision, body, and gravity but in the supine by only two (body and vision) (since gravity is now acting orthogonally to this plane). Therefore, by geometry, the VE is predicted to get larger in the supine condition (as shown in the inserts above Fig. 5A). Figure 5A plots the VE as a function of bed rest session for the supine (green line) and upright (red line) conditions for the PU. A repeated measures analysis of the VE for supine and upright participants before they started their bed rest exposure found a significant effect of body orientation F(1,8) = 5.323, *p* = .050, ɛ_p_^2^ = 0.400; while for post bedrest session 1 the VE upright and supine were not significantly different F(1,8) = 0.004, *p* = .952, n.s., ɛ_p_^2^ = 0.004.

Similar to the VE, the gravity effect (GE) is defined as the difference in the perception of upright when only the orientation of gravity is tilted relative to the participants (here from left to right without visual cues to upright). If the contribution of the body cue were to become larger as a consequence of bedrest, then the relative contribution of the gravity cue should decrease, and the GE would be expected to correspondingly decrease. Figure 5B plots the GE as a function of bedrest session for both the PU (orange line) and the SVV (green line). A repeated measures analysis of the GE for side-down participants before and after bed rest found no significant effect when measured by PU; F(2,16) = 3.421 *p* = .058 n.s., ɛ_p_^2^ = 0.300, or when measured by SVV; F(2,16) = 0.738 *p* = .494 n.s., ɛ_p_^2^ = 0.084.

### 3.3 The weighted vector sum model

**Fig. 6:**
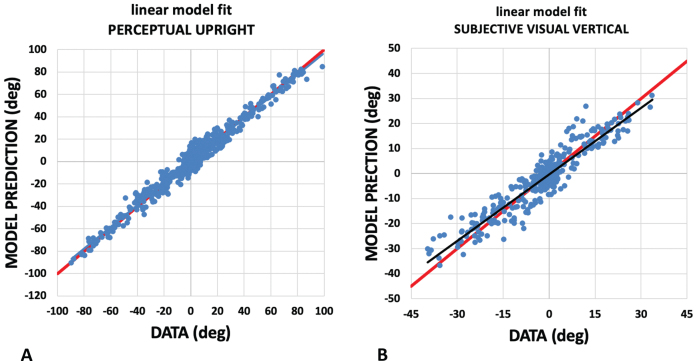
Assessing the linear weighted vector sum model. The weighted vector sum model described, models the PU and SVV as a weighted linear vector sum of vectors aligned with gravity, the body, and the visual display. Here we plot the model’s prediction (vertical axis) against participant responses (horizontal axis) for the PU (A) and SVV (B). For PU, the plot has a slope of 0.97 with a y intercept of 0.03 and for the SVV the plot has a slope of 0.89 with a y intercept of –0.31. These fits are drawn in black. Perfect responses are drawn in red.

Although the SVV and PU showed no significant change with bed rest this does not necessarily mean that the weightings assigned to the various cues remained constant over the testing period. Rather, the pattern of responses to variations in the orientation of the body and visual cues can be used to probe how the cues are combined to provide a perception of the direction of up. Both the PU and SVV can be modeled as a linear weighted sum of three vectors pointing in the directions signaled by visual, gravity and body cues as follows [21]:

(3) $$\begin{array}{cc} & \mathrm{up}=\mathrm{vision}*\mathrm{weigh}t_{\mathrm{vision}}+\mathrm{body}*\mathrm{weigh}t_{\mathrm{body}}\\ & \;\;\;\;\;+\mathrm{gravity}*\mathrm{weigh}t_{\mathrm{gravity}}+\mathrm{bias} \end{array}$$

where vision, body, and gravity are vectors in the appropriate directions associated with each cue, each with its own weighting expressed relative to the others. The weighted vector sum model has proven sufficient to explain a number of cue integration results [21, 25] although more sophisticated models exist [35, 36]. We separated the directions indicated by each cue experimentally so that the relative magnitudes of the weights could be calculated and expressed as a percentage adding up to 100%. The SVV and PU measured in the upright, left-side-down and right-side-down conditions (thus varying the direction of gravity relative to the body) and with different visual backgrounds (thus varying the direction of the visual cues to upright) were fitted to equation 3. The three-vector model was fitted using a non-linear least-squares optimization for each probe-body orientation condition using Python’s SciPy minimization function configured to use the Broyden-Fletcher-Goldfarb-Shanno algorithm. Figure 6 shows the effectiveness of the model in predicting the data. This provided the relative weighting of vision, gravity, and body cues contributing to the SVV and PU for each pre- and post-bed rest data collection session. For the in-bed data, only on-side and supine data were available for the PU analysis. Since only the on-side data were available for the SVV in the in-bed phase, modeling could not be reliably performed for these conditions.

**Fig. 7:**
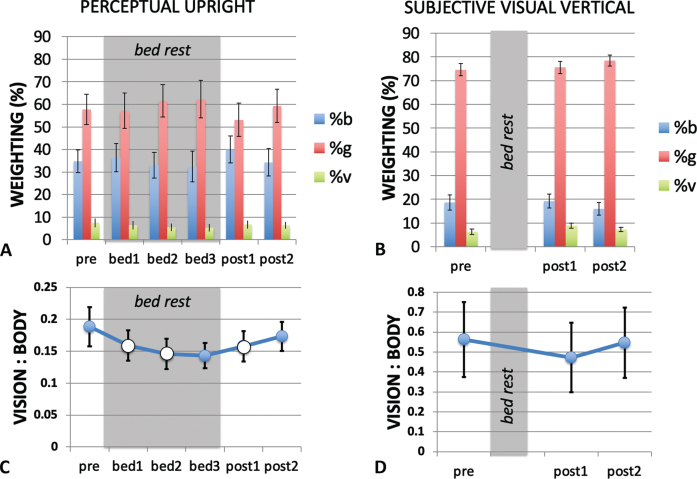
Modeling the PU and SVV using a weighted vector sum model. The relative weighting of the body (b), gravity (g) and visual (v) cues are shown for the PU (A) and SVV (B) for each measurement session. The in-bed sessions are indicated by the grey shading. (C) and (D) show the ratio of visual to body weighting for each measurement session. When considering the critical sessions pre, bed3 and post2 only, the PU the vision-to-body ratio (C) declined significantly during HDBR, returning to pre bed rest conditions after bed rest ended. (Data from critical session are filled in (C) while data from non-critical sessions are open.) The vision-to-body ratio for the SVV (D) showed no change from pre bed rest to post bed rest.

Repeated measures analysis of vector weighting for six conditions (6; pre, bed1, bed2, bed3, post1, post2) was performed for the body, gravity and vision weightings computed using both the PU probes and similarly for the SVV weightings for three conditions (3; pre, post1, post2). For the PU there was no significant effect of session on the weightings of body F(5,40) = 1.513, *p* = .569 n.s., ɛ_p_^2^ = 0.042, or gravity F(5,40) = 1.7954, *p* = .136 n.s., ɛ_p_^2^ = 0.183. However, there was a significant effect of session on the vision weighting F(5,40) = 3.397, *p* = .012, ɛ_p_^2^ = 0.042. Post hoc t-tests with Bonferroni correction showed no significant pairwise comparisons. For the SVV there was no significant effect of session on the weightings of body F(2,8) = 0.890, *p* = .430 n.s., ɛ_p_^2^ = 0.100, gravity F(2,8) = 1.514, *p* = .250 n.s., ɛ_p_^2^ = 0.159, or vision F(2,8) = 2.074, *p* = .158n.s., ɛ_p_^2^ = 0.206. The relative weightings are plotted in Fig. 7A and B.

**Fig. 8:**
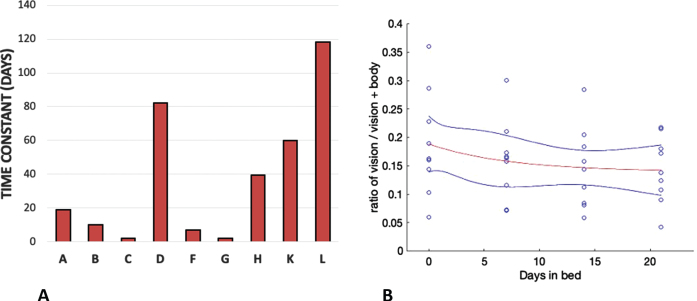
The time constants of exponential decline of the ratio of vision to vision+body weighting for each participant (A) and the exponential fitted through the whole data set (red line) with a time constant of 7.28 days (B). The blue lines show 95%confidence limits.

The changes in relative weighting between in-bed and pre- and post- bed rest conditions were further examined using the ratio of the vision-to-body cue weights (Fig. 7C and D). Exponential functions of the form

(4) $$\mathrm{ratio}=\mathrm{rati}o_{\Delta }e^{-t/-T}+\mathrm{rati}o_{\infty }$$

were fitted using Matlab v2018b to each of person’s vision:body ratio for the duration of their stay in HDBR (21 days) to assess the time constant of change from pre-bed levels. The time constants (tau) for each participant are shown in Fig. 8A. The mean time constant of decay for an exponential plotted through the entire data set was 7.28 days and is shown in Fig. 8B. Although the paucity of data points per individual introduces some uncertainty in terms of the fits, the fits do show a significant time constant of several days and considerable variability across participants. Given the range of time constants analysis of the vision:body ratio was restricted to pre bed rest, the final bed rest data point (bed rest session 3) and the final post bed rest recover data point (post bed rest session 2).

The orientation of the long axis of the body (the body cue to orientation) always indicates that gravity is above the head independent of the orientation of the body. The ratio of vision to body cues that determine the PU declined during bed rest dropping from a ratio of 0.19 (pre bed rest, i.e., baseline) to 0.14 in bed rest session 3 (corresponding to the longest duration of HDBR), before returning to 0.17 by post bed rest session 2 (the longest duration post HDBR). This observation was confirmed through a Friedman Test χ^2^(2) = 8.667, *p* = .013 for pre bed rest, bed rest session 3 and post bed rest session 2. Post-hoc analysis using the Wilcoxon signed-rank test with Bonferroni correction confirmed that the vision-to-body ratio declined from pre-bed rest to in bed session 3 *Z* = –2.547, *p* = .033 while the other condition pairs were not significantly different (Fig. 7C). When the analysis is extended to include data from all six sessions, this significance is lost χ^2^(9) = 10.587, *p* = .060. For the SVV, a Friedman Test χ^2^(2) = 1.750, *p* = 0.417 n.s. was performed for pre bed rest, post bed rest session 1 and post bed rest session 2 (Fig. 7D). A non-parametric test was used here given the ratio nature of the data and the moderate skewness in the pre-bed rest data for OChaRT and the highly skewed nature of the SVV data. We concentrated on these data points as they correspond to the longest period of adaptation to, and recovery from, HDBR as measured by OChaRT and SVV in this study.

### 3.4 Variance

Individual participant responses were fit with either a hyperbolic tangent (SVV, Equation 1) or a product of two hyperbolic tangents (PU, Equation 2). The t values in these equations were converted to degrees and used to identify the *σ* for the best-fit Gaussian approximation to the hyperbolic tangent. The variance (*σ*^2^) of the PU and SVV estimates the variability associated with each of the cues. The PU was collected under conditions when either (a) only body cues were available (supine participant with a grey background), (b) when body and visual cues were available to influence the perception of the probe character (supine participant with a visual background aligned with the body), (c) when just body and gravity cues were available (upright with a grey background), and (d) when all cues were available (upright with a visually upright background). The SVV was collected only under conditions (c) and (d) because when supine, gravity was orthogonal to the display and therefore the line could not be aligned with gravity using our paradigm.

**Fig. 9:**
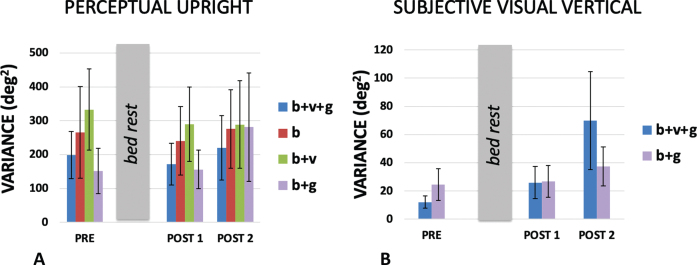
Variances for the (A) PU and (B) SVV for conditions in which cues present were aligned. The variances were obtained under conditions in which only certain combinations of body (b), visual (v) and gravity (g) cues were available as indicated in the legend on the right of each bar chart (see text). Standard errors are shown.

The mean variances obtained under each of these conditions are plotted in Fig. 9. We might expect variance to decrease as more cues became available however we acknowledge that our measure is based on a fit through rather few data points. Figure 1 shows that in many cases the psychometric curve was based on only four or five points. A repeated measures analysis of PU variances was conducted for session (3: pre, post1, post2) x cue (4: *b* + v+g, *b* + v, *b* + g, b). Mauchly’s test of sphericity was violated for session χ^2^(2) = 8.412, *p* = .015, and DOF for session was corrected using Greenhouse-Geisser. There was no effect of session F(1.177,9.415) = 0.363, *p* = .596 n.s., ɛ_p_^2^ = 0.043, there was no effect of cue F(3,24) = 1.616, *p* = .212 n.s., ɛ_p_^2^ = 0.168, and there was no interaction effect F(6,48) = 0.459, *p* = .835 n.s., ɛ_p_^2^ = 0.054.

A repeated measures analysis of SVV variances was conducted for session (3: pre, post1, post2) x cue (2: *b* + v+g, *b* + g). Mauchly’s test of sphericity was violated for session χ^2^(2) = 14.985, *p* = .001, and DOF for session was corrected using Greenhouse-Geisser. There was no effect of session F(1.062) = 4.957, *p* = .053 n.s., ɛ_p_^2^ = 0.383. The near significance of this test is probably accounted for by the large *b* + v+g variance measured on the last day which may have been caused by other factors (POST 2, Fig. 9b). There was no effect of cue F(1,8) = 0.507, *p* = .497 n.s., ɛ_p_^2^ = 0.060, and there was no interaction effect F(2,16) = 1.300, *p* = .300 n.s., ɛ_p_^2^ = 0.140.

## 4 Discussion

Detailed analysis of the relative weighting of the cues that contribute to the PU revealed a decrease in the weighting of vision relative to the body after 21 days as a consequence of HDBR which recovered to pre bed rest levels within 60 days (Fig. 7C). This significant finding is based on the analysis of the data from the three critical sessions: pre-bed rest, at the end of bed rest, and after long-term recovery from bed rest. When all sessions were included in the analysis this significant result was attenuated to a strong trend. No significant changes in the effect of vision on the SVV were found or for the effect of gravity on either the PU or the SVV. This is in agreement with the only other study to look at the effect of HDBR on the perception of orientation which also reported no effects on the SVV [39].

### 4.1 Comparison between HDBR and long-duration spaceflight

Head down bed rest has often been used as an analog for long-duration spaceflight [43, 45, 50]. Here we are interested in its effects on perceived self-orientation. HDBR has been shown to affect balance even within 14 days (see [48] for a review) but how much of this may be due to muscle issues and how much to sensory changes is unclear. HDBR causes muscle weakening especially in the absence of exercise [30] which is likely to cause standing instability that may in turn mask or enhance the effects of any sensory consequences such as the changes in sensory weightings reported here.

Clark [11] provides an excellent review of studies on the effect of microgravity on the perception of static tilt. Small static roll tilts tend to be overestimated post-flight [16]. This has been taken to indicate “vestibular release” and contributed to the prolonged debate on the tilt-translation hypothesis [44] in which tilt following space flight may be interpreted as translation. Harris et al. [25] is the only study to date to look at the influence of visual cues on static orientation. They noted two effects of exposure to long-duration microgravity on the perception of upright. Because going into microgravity removes the influence of gravity from the long axis of the body, Harris et al. [25] predicted that the effect of vision should increase. This follows from the simple geometry summarized in the inserts to Fig. 5A. Harris et al. [25] did not find this expected increase in the VE during microgravity exposure implying that a compensation had taken place to maintain the ratio of vision to body cues.

HDBR appears to do the same thing. Fig. 5A shows a decline in the influence of vision over the course of the 21 days of bed rest and this decline is reflected in declines in both the raw percentage vision in the weighted vector sum model (Fig. 7A) and the ratio of vision to body weightings (Fig. 7C). The time constant of decline in bedrest appeared to be much slower than seems to be the case in space. Here the average time constant to reach asymptote was 7.28 days taking 21 days to reach a level that was significantly different from pre-HDBR levels. There is no directly comparable measure of the time course in space but already by day 10 significant effects were recorded by Harris et al. [25].

A second effect that Harris et al. [25] noted as result of exposure to long-duration microgravity was that variance of the SVV increased significantly upon return to a 1G environment from microgravity. No such significant effects were found here upon recovery from HDBR, although there was a strong trend (*p* = 0.053) for the variance in the SVV measures associated with HDBR to also increase post bed rest compared to pre bed rest measurements (Fig. 9B). There are of course substantive differences in timings between the two studies. Here, post bed rest measurements occurred 14 days after the HDBR period, while in Harris et al. [25] post flight measurements took place on average 12 and 130 days post flight. The microgravity exposure in Harris et al. [25] lasted 180 days on average, compared to the 21 days of HDBR.

### 4.2 Cue weightings

In order to analyze the mechanism of change in vision we fitted our data with a weighted vector sum model. This model has been found to fit data from a wide range of body orientations [6] although additional assumptions can be introduced to allow for changes in the sensitivity of the utricle and saccule with orientation [33]. The analysis revealed a significant pattern of change under HDBR. The ratio of the weighting of vision-to-body declined during HDBR and returned to pre bed rest values upon return to normal conditions (Fig. 7C). Harris et al. [25] reported a similar finding in which for 100%of astronaut subjects, the vision: body ratio declined relative to pre-flight testing both early in flight and long after return to a 1G environment. Here we find a similar decline during HDBR, with participants still not showing the normal increase in visual effect on lying supine even by their last post bed rest measurement (Fig. 5A). All these observations can be compared to those of a baseline control group (data reported in [25]) in which the PU, SVV, and weightings remained constant during a whole year of testing. This suggests that both during HDBR and in microgravity, participants re-weight their vision-to-body weightings so as to not inflate the influence of vision (the VE) under these conditions. That is, the weightings were influenced by the context under which the measurements were taken. That no effects of HDBR were found in the SVV here or in a previous study [39] may be related to the relatively minor contribution of vision in determining the SVV.

Interestingly, there was no change in gravity effect (Fig. 5B) suggesting that the weighting of gravity relative to the body was not significantly altered by HDBR and thus suggesting that the weighting of the body and gravity cues both increased relative to vision. An increase in the weighting of the body might underlie the observation that after 21 days of HDBR participants feel that they are lying horizontal rather than 6° head down [27].

### 4.3 Significance

Reducing the weight placed on the visual cue and instead relying more on the body cue may be an adaptive strategy in space or during HDBR in which the rules of statistical optimality may be overridden. Under normal conditions the visual, body and gravity cues to upright are in agreement most of the time. But in the environment of a space station, the visual cues no longer signal a consistent up direction. Similarly, in HDBR the visual cues to upright also do not line up with, and in fact are constantly almost orthogonal to, the body. In both HDBR and under microgravity it may be an optimal strategy to downplay the visual cues and tend to rely more on the idiotropic vector as the dominant up reference. That is, the rules of statistical optimality can be overridden by the principle of robustness when cues consistently are not in agreement [29].

A limitation of this study was that, for administrative reasons, the study was performed on a relatively small and entirely male population who were self-selected based on availability and were fit and young. This makes it hard to generalized to the general population. Furthermore, the study was limited to 21 days of HDBR which makes it hard to compare to space flights that may last more than a year. The high degree of variance found for our measures (Fig. 9) may have hidden some effects. For example, females rely more on external visual cues to in many spatial orientation tasks [3, 37] and are less susceptible to visual-vestibular conflict [52] suggesting that they may be less influenced by gravity cues to upright [5]. We cannot predict the female response to HDBR from the present study. This is an interesting direction for future research.

### 4.4 HDBR as an analog for long duration spaceflight

HDBR is an attractive analog for long-duration spaceflight which has been shown to be highly effective in mimicking many of the physiological changes associated with microgravity exposure [43, 50]. Notwithstanding the inconvenience for the participants, HDBR is substantially less expensive, less dangerous, and provides the potential for a much wider participant pool and more sophisticated experimental apparatus than is possible with astronauts. It would be difficult and expensive to ship an fMRI machine to station, for example. That being said, it is essential to ensure that the analog is accurate for the physical/sensorimotor/perceptual system under study, and to understand the limits of the analog. This study is the first to measure the perceptual upright during and following HDBR and demonstrates for the first time that for the perception of static self-orientation using two different probes, similar effects are associated with both HDBR and long-duration spaceflight using essentially identical methodologies. In particular, (a) neither HDBR nor long-duration spaceflight seems to impact the SVV or the PU, (b) long-duration spaceflight increases the variance of SVV and a comparable trend was found after HDBR and (c) decomposing the contributing factors of the PU into a weighted vector sum model showed a significant decrease in the ratio of vision-to-body weighting during HDBR relative to pre-HDBR levels that recovered post bed rest. Long duration spaceflight also evoked a decrease in the ratio of vision-to-body cue weighting within the first 10 days of flight that re-emerged late post flight (mean 130 days). Detailed assessment of the time course of these changes in space and in HDBR may provide important information about the nature of adaptation to microgravity exposure and to addressing safety concerns during the initial adaptation phase. In summary, HDBR appears to be a useful analog for the study of the perception of self-orientation during long-duration space flight.
